# The relationship between long working hours and depression among first-year residents in Japan

**DOI:** 10.1186/s12909-018-1171-9

**Published:** 2018-03-27

**Authors:** Ryoko Ogawa, Emiko Seo, Takami Maeno, Makoto Ito, Masaru Sanuki, Tetsuhiro Maeno

**Affiliations:** 1 Center for Medical Education and Training, University of Tsukuba Hospital, 2-1-1 Amakubo, Tsukuba, Ibaraki 305-8576 Japan; 2 Graduate School of Comprehensive Human Sciences, Faculty of Medicine, University of Tsukuba, 2-1-1 Amakubo, Tsukuba, Ibaraki 305-8576 Japan; 3 National Hospital Organization, Kasumigaura Medical Center, 2-7-14 Shimotakatsu, Tsuchiura, Ibaraki 300-8585 Japan

**Keywords:** Medical residency, Resident, Work hours, Depression, Mental health

## Abstract

**Background:**

In Japan, some residents develop mental health problems. In previous studies, it was reported that long working hours might be a cause of stress reaction such as depression. There were some reports that compared residents with 80 or more working hours with those with less than 80 working hours. However, many residents are practically detained for extra-long time, designated as 100 h or more per week, for medical practice, training, self-study, etc. There have been few reports on extra-long hours of work. This study evaluated the working environment and the amount of stress experienced by first-year residents, and examined the relationship between long working hours and depression, especially in the group of extra-long working hours.

**Methods:**

The study included 1241 first-year residents employed at 250 training hospitals in 2011. A self-report questionnaire was administered at the beginning of the residency and 3 months later to collect data on demographics, depressive symptoms, and training conditions (e.g., duration of work, sleep, disposable time, and night shift). Depressive symptoms were rated using the Center for Epidemiologic Studies Depression Scale.

**Results:**

The mean duration of work per week was 79.4 h, with 97 residents (7.8%) working 100 h or more. At 3 months, clinically significant depressive symptoms were reported by 45.5% of residents working 100 or more h per week, which proportion was significantly greater than that for respondents working less than 60 h (*P* < 0.001). Multivariate logistic regression analysis showed that a working week of 80 to 99.9 h was associated with a 2.83 fold higher risk and 100 h or more was associated with a 6.96-fold higher risk of developing depressive symptoms compared with a working week of less than 60 h.

**Conclusion:**

Working excessively long hours was significantly associated with development of depressive symptoms. Proper management of resident physicians’ working hours is critical to maintaining their physical and mental health and to improve the quality of care they provide.

## Background

Medical profession is stressful and is associated with a high prevalence of depression, burnout, and mental health problems [1–3]. Resident physicians are at a higher risk of developing stress than are more experienced physician due to their recent transition from student to professional life and lack of interpersonal and communication skills, medical knowledge, and experience [4, 5]. A long time is required for the resident physician as a worker and trainee on the job training. Because they are still inexperienced as physicians, they need time to take lecture and training to do self-study in addition to the time to perform a physical exam. It has been reported that long working hours stresses resident physicians as independent stress factor and contributes to depressive symptoms [1–4]. The prevalence of depression among residents is variable, with reports ranging from 7%-35%, however most studies have found the rate of depression among residents to be higher than that of the general public (4%-5%) [6].

Such working conditions constitute a major source of chronic stress, which is associated with physical and mental health problems, such as depression and, in extreme cases, suicide [7, 8].

Appropriate management of resident working time is essential to ensure the quality of the medical services they provide. Fahrenkopf AM et al. reported that depressed residents made 6.2 times as many medical errors per month as residents who were not depressed [9]. Decreased performance resulting from overwork and exhaustion can increase the risk of introducing medical errors [10, 11].

It is imperative that residency hospitals ensure a training environment that maintain the health of their residents. In the previous study, Yoshino S et al. reported that the training environment of more than 80 work hours per week significantly causes the depressive symptom of the resident [12]. In the United States, the Accreditation Council for Graduate Medical Education(ACGME) restricted work and training time to less than 80 h per week in 2003, because long working hours is detrimental to the health of resident and increases the risk of medical error [13]. However, in many hospitals, it is very difficult to restrict within 80 h including training times. Maeno et al. reported in 2004 that Japanese residents worked a means 85.0 h per week and residents worked over 100 h per week is 16.4% [4]. and Landrigan CP et al. reported that first year residents worked mean 84.9 h per week in intensive care units [11] and Gelfand DV et al. reported in 2004 that resident work hours per week decreased from 100.7 h to 82.6 h since ACGME restricts resident working hours in the surgery department, but this is still over 80 h [14]. As such, we think that a longer time is required for many resident physicians effectively for medical practice, training, self-study, etc. The training program director had to manage working hours including training time and to maintain the conditions of residents, so in this situation clarified pratical and realistic minimum requirement to protect resident’s mental health is needed. There have been few reports on extra-long hours of work that exceeded 80 h. In this study, We conducted a large-scale survey of all teaching hospitals in Japan and revealed the working environment of the first year residents, including working hours, sleeping hours, disposable times, and the number of days off work. And We also estimated the relationship the work hours including extra-long working hours and the depressive symptom.

## Methods

### Subjects

We contacted 853 postgraduate training hospitals across Japan using the REIS (Residency Electronic Information System), which is the Japanese government database of hospitals and programs providing clinical training. A total of 250 teaching hospitals were willing to cooperate. The study involved surveys carried out at two time points; at the beginning of residency and 3 months later. These time points were chosen with a previous study reporting that the highest risk of depression occurs during the first months of residency [4, 15]. The study population included 1241 first-year residents who responded to the two self-administered questionnaires in 2011.

### Measurement

In order to comprehensively assess the mental health problems, this study evaluated the following parameters using the self-report questionnaire.

1. Depressive Symptoms

We used the CES-D Scale (Center for Epidemiologic Studies Depression Scale) to evaluate depressive symptoms. The CES-D Scale was developed as a case-finding tool to screen for depressive symptoms in the general population by the US National Institute of Mental Health and is widely used as a self-report scale to measure symptoms associated with depression [16, 17].

2) Work Environment

Parameters related to the work environment included average number of hours spent at the hospital (including both clinical practice and educational activities) per day during the work week and weekends, night shift, the day after a night shift; number of hours of disposable time per day (time available for personal disposal, excluding time for work, eating, sleeping, and other necessities); number of hours of sleep; and number of days off work.

We have calculated mean number of working hours per week using the following formula.

#### Mean working time

Based on previous studies [4, 17–20], working time per week was calculated using the following equation, involving three components: mean working time on weekdays, mean working time on weekends, and monthly number of night duties.

$$ \mathrm{Hence},\mathrm{the}\ \mathrm{mean}\ \mathrm{weekly}\ \mathrm{working}\ \mathrm{time}=\left[5\times \left(\mathrm{mean}\ \mathrm{working}\ \mathrm{time}\ \mathrm{on}\ \mathrm{weekdays}\right)\right]+\left[2\times \left(\mathrm{mean}\ \mathrm{working}\ \mathrm{time}\ \mathrm{on}\ \mathrm{weekends}\right)\right]+\left[7\times \left(\mathrm{number}\ \mathrm{of}\ \mathrm{night}\ \mathrm{duties}\ \mathrm{per}\ \mathrm{month}/30\right)\times \left(24-\mathrm{mean}\ \mathrm{working}\ \mathrm{time}\ \mathrm{on}\ \mathrm{weekdays}\right)\right]. $$

The mean weekly working time was defined as the sum of five times the mean daily working time on weekdays, plus two times the mean daily working time on weekends, plus the mean working time during night duties.

3) Demographics

The first survey, administered at the beginning of the orientation program, included age, gender, type of hospital (university or non-university).

### Study design

As mentioned above, this study included two surveys. The questionnaire forms were distributed to residents via the residency program director. The received responses were anonymized for data processing by assigning different ID numbers for the first and second questionnaire responses from each individual resident, although the data were traceable by cross-referencing the ID numbers.

### Statistical analysis

1. Association between the Number of Working hours per Week, Sleep, and Disposable time

Univariate analysis (Student’s *t*-test) was used to evaluate the association between the number of working hours per week calculated as described above, and the number of hours available for sleep and disposable time, respectively.

2) Risk Factors for Depression

In order to evaluate the association between work conditions and the onset of depressive symptoms, residents who had depression (i.e., residents with CES-D scores ≥16) at baseline (i.e., at the time of the first survey), were excluded from analysis.

A CES-D cut-off score of 16 was indicative of depression. Univariate analysis was used to identify risk factors for depression. Specifically, Student’s *t*-test was used for continuous variables (number of working hours per week, hours available for sleeping, and disposable time), and the chi-square test was used for categorical variables (gender, and type of hospital). In addition, the association between the number of working hours per week and CES-D score was assessed by stratifying the number of working hours per week into six groups (0–59, 60–69, 70–79, 80–89, 90–99, and ≥ 100 h). Next, potential risk factors for depression identified in the univariate analysis were included in a multivariate logistic regression model (forced entry method) to determine odds ratios (ORs) and 95% confidence intervals (CIs). In the multivariate logistic regression analysis, depression status (CES-D score < 16 or ≥ 16) was the dependent variable, and gender, age, and number of working hours per week were independent variables. The number of working hours per week was categorized into four groups: 0–59, 60-79, 80–99, and ≥ 100 h.

The SPSS statistics software version 22.0 for Windows (IBM Japan, Ltd., Tokyo, Japan) was used for statistical analysis.

### Ethical considerations

The study was approved by the Ethics Committee of the Faculty of Medicine of the University of Tsukuba. The purpose of this study and measures to ensure secure data management were stated on the first page of the questionnaire. We also explained to potential participants that their involvement in the study was purely voluntary. Results were analyzed separately from personal information in order to allow for anonymity and confidentiality of personal information.

## Results

### Demographics

Among 2935 residents employed at 250 participating teaching hospitals, 1754 responded to at least one of the surveys. Complete responses to both surveys were obtained from 1241 (42.3%) residents. Of these, 495 (39.9%) residents undergoing training at university hospitals. (Table 1).

**Table 1:** Participant characteristics (*n* = 1241)

Age, mean ± SD, y	26.0 ± 2.9
Male Sex, n (%)	812 (65.4%)
Hospital type	
University hospital, n (%)	495 (39.9%)
Other teaching hospital, n (%)	746 (60.1%)
Mean number of work hours per week, mean ± SD	79.4 ± 15.0
on weekdays, mean ± SD	12.2 ± 2.3
on weekends, mean ± SD	4.3 ± 3.2
80 or more hours of work per week, n (%)	554 (44.6%)
100 or more hours of work per week, n (%)	97 (7.8%)
Mean number of hours for sleep per day, mean ± SD	5.8 ± 0.9
Less than 6 h for sleep per day, n (%)	409 (33.0%)
Mean duration of disposable time per day, mean ± SD, hours/day	2.8 ± 1.6
Less than 2 h of free time, n (%)	244 (19.7%)
Number of night shifts per month, n (%)	3.6 ± 2.1
Working schedule after night shifts (*n* = 1065)	
No change, n (%)	726 (68.2%)
Shorter working day, n (%)	291 (27.3%)
Rest day, n (%)	48 (4.5%)
Mean number of days off work per month, mean ± SD	4.7 ± 3.9
Number of calls during overtime duty per month, mean ± SD	3.0 ± 6.1

### Work conditions

The mean duration of work per day was 12.2 h during working weeks and 4.3 h during weekends. The mean duration of work per week was 79.4 h, 554 residents (44.6%) worked 80 h per week or more and 97 residents (7.8%) worked 100 h per week or more. Residents had an average of 3.6 night shifts per month. In regards to the schedule of the day following a night shift, 68.2% of residents had a normal work schedule. The mean number of days off from work was 4.7 per month, and residents received an average of 3.0 calls from hospital staff after office hours (Table 1). The mean duration of work per week at non-university hospitals was 81.4 h, which was longer than that at university hospitals (76.4 h, *p* < 0.001).

**Fig. 1:**
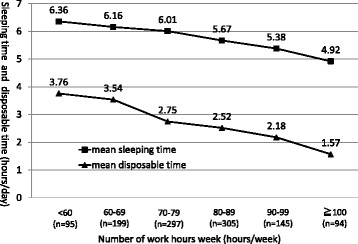
Association between the number of work hours per week and number of hours for sleep per day or amount of disposable time per day (*n* = 1135). ■:the mean number of hours for sleep per day ▲:the mean duration of disposable time per day. The graph shows the mean number of hours for sleep per day and the mean duration of disposable time per day at the 3 months later of first-year residency analyzed by stratifying the number of working hours per week into six groups (0–59, 60–69, 70–79, 80–89, 90–99, and ≥ 100 h)

The mean number of hours available for sleep was 5.8 h per day, and 409 residents (33.0%) slept less than 6 h daily. The mean disposable time was 2.8 h per day, and 244 residents (19.7%) had less than 2 h of disposable time on a daily average. Residents with longer working hours had a shorter duration of sleep and less disposable time (Fig. 1).

### Depressive symptoms

In the evaluation of depressive symptoms, 86 respondents had missing data and 182 respondents whose baseline CES-D scores were 16 or above were excluded, leaving 973 residents for analysis. After 3 months of residency, 22.6% of residents exhibited newly developed depressive symptoms. Chi-square analysis of the association between the onset of depressive symptoms and working hours, gender, type of hospital, habits showed that residents who worked for 80 h or more per week had a significantly higher incidence of depression than those who worked for less than 80 h (28.5% and 17.3%, respectively; *P* < 0.001). The analysis using the *t-*test showed that residents experiencing new-onset depressive symptoms had significantly fewer hours available for sleep and less disposable time (*P* = 0.010 and *P* < 0.001, respectively, Table 2).

**Table 2:** Working environment and new-onset depressive symptoms (*n* = 973)

	New-onset depressive symptoms (+) (CES-D ≥ 16)	New-onset depressive symptoms (−) (CES-D < 16)	*P* value
Age, mean ± SD, y^a^	26.0 ± 2.98	26.0 ± 2.91	0.751
Male sex, n (%)^b^	142 (64.5%)	502 (66.8%)	0.297
University hospital, n (%)^b^	85 (38.6%)	292 (38.8%)	
Other educational hospital	135 (61.4%)	461 (61.2%)	1.000
Number of hours for sleep per day, mean ± SD,^a^	5.68 ± 0.95	5.81 ± 0.87	0.010
Disposable time, mean ± SD, hours/day^a^	2.35 ± 1.49	2.87 ± 1.56	< 0.001
Number of work hours per week^b^			
< 60 h (*n* = 84)	9 (10.7%)	75 (89.3%)	
60 to < 80 h (*n* = 429)	80 (18.6%)	349 (81.4%)	
80 to < 100 h (*n* = 383)	96 (25.1%)	287 (74.9%)	
≥ 100 h (*n* = 77)	35 (45.5%)	42 (54.5%)	< 0.001

*CES-D* Center for Epidemiologic Studies Depression Scale

^a^*t*-test

^b^ Chi-square test

**Fig. 2:**
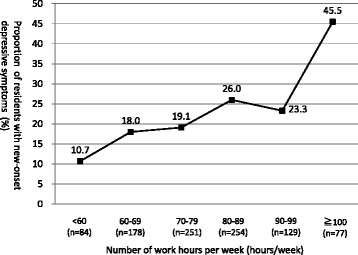
Association between the number of work hours per week and new-onset depressive symptoms (*n* = 973). The graph shows the proportion of new-onset depressive symptoms at the 3 months later of first-year residency analyzed by stratifying the number of working hours per week into six groups (0–59, 60–69, 70–79, 80–89, 90–99, and ≥ 100 h)

In consideration of the relationship between working hours and depressive symptoms, 25.1% of residents who worked 80 to 99 h per week and 45.5% of those who worked for 100 h per week or more experienced newly developed depressive symptoms (Table 2). Analysis stratified by 10-h intervals showed that the proportion of new-onset depressive symptoms increased with an increasing number of working hours (Fig. 2). This tendency was remarkable especially in cases where the working hours exceeded 80 h.

By multivariate logistic regression analysis, the OR for depression in those who worked 80–99 h per week and 100 h per week or more was 2.83 and 6.96, respectively, with those who worked less than 60 h as the reference group (Table 3).

**Table 3:** Association between the number of work hours per week and new-onset depressive symptoms

		Odds ratio	95%CI
Mean number of work hours per week	< 60 h	1.00	
	60 to < 80 h	1.9	0.92-4.00
	80 to < 100 h	2.83	1.36-5.88
	≥100 h	6.96	3.05-15.90
Age		1.02	0.96-1.07
Sex^a^		1.15	0.84-1.60

Base on a binomial logistic regression analysis where the dependent variable was the presence of new-onset depressive symptoms and the independent variables were mean number of work hours per week (4 categories), age, and sex

*Abbreviations*: *CI* confidence interval

^a^Coded as: 0 = male; 1 = female

## Discussion

In this study, We clarified the working environment of the first year residents and the relationship between long working hours and depression. First year residents in Japan worked long times of mean 79.4 h per week. By the third month of residency, 22.6% of the residents had newly developed depressive symptoms. As a result of analyzing working hours per week every 20 h by multivariate logistic regression analysis, the odds ratio was 2.83 in the group of 80–99 h work hours per week and 6.96 in the group of 100 h or more work hours per week. This result proved that the risk of depression was increased extremely.

### Work environment

On average, residents worked 79.4 h per week, and clinically mild or significant depressive symptoms were newly observed in 28.5% of residents worked 80 h or more per week. These results suggest that a considerable proportion of residents probably had mental health problems requiring medical treatment. In addition, residents slept an average of 5.8 h per day and had a daily average of 2.8 h at free disposal. These findings indicated a prevailing lack of rest among residents. According to the *2011 Survey on Time Use and Leisure Activities* released by the Ministry of Internal Affairs and Communications of Japan [21], younger people aged 25 to 29 years, on average worked for 8.8 h, slept for 7.3 h, and had an average of 4.1 h free at their disposal. These results suggest that resident physicians have a longer and more intense work schedule than their age-matched peers in other professions. The fact that 68.2% of the residents had a normal work schedule on the day following a night shift suggests that a majority of the residents occasionally worked for more than 24 h consecutively. Extended work shifts lasting for 24 h or more are associated with higher rates of attentional failure during night work hours compared with shifts of consecutive 16 h or fewer [10]. In 2003, the Accreditation Council for Graduate Medical Education (ACGME) set a 24-h limit on continuous duty for residency programs in the United States. The ACGME also established a 16-h limit for first-year residents in 2011 [13, 22]. In addition to the amount of working time per week, the duration of continuous duty is an important aspect for residency programs.

### Relationship between work environment and depression

By the third month of residency, 22.6% of the residents had newly developed depressive symptoms. Our study found a considerably high incidence of depressive symptoms among resident physicians, in spite of depressive symptoms at baseline being 16.0%. During the first few months of residency, physicians face particularly strong stressors, such as the change from student to professional life, exposure to new interpersonal relationships and work environments, and anxiety arising from the gap between high professional demands and insufficient skills and experience [3, 23, 24].

In our study, 25.1% of residents working 80–99 h per week and 45.5% of those working for 100 h per week or more developed new symptoms. A high workload can be a major source of stress that leads to depression. In a study of Canadian residents [25], time pressure was the most frequently reported source of stress experienced by 70% of the residents surveyed. Proper work management and supervision are key in maintaining the mental and physical health of residents, because long working hours are an independent risk factor for depression [26]. Working long hours is also associated with anxiety, low sleep quality, and coronary artery disease [27]. Our study demonstrated a significantly higher incidence of depressive symptoms among residents working 80 h per week or more, a finding consistent with the ACGME’s recommendations for an 80-h working week [20]. Our results, as well as the recommendations from ACGME, indicate that the administrators of residency programs should consider an 80-h work week as the upper limit for healthy residency training.

Our study revealed that the longer the working hours, the more the number of residents who developed depressive symptoms and extra-long working hours per week (≥100 h/week) was associated with approximately 7 fold higher risk of developing depressive symptoms compared with a working week of less than 60 h. In our study, the number of residents who worked 80 h or more per week was 554 (44.6%) and who worked 100 h or more was 97 (7.8%), so very many residents had more than 80 work hours per week. We think that the working times of the resident is necessary to be less than 100 work hours per week including training times in any busy environment from this result.

Our study shows a negative correlation between the number of working hours per week and the number of hours available for sleep, which is consistent with a previous study [18, 26, 27]. A case-control study of Japanese patients admitted for acute myocardial infarction showed that daily sleep duration of less than 5 h was associated with a 2.5-fold increase in the risk of developing acute myocardial infarction [28]. Chronic sleep deprivation can increase the risk of developing physical illness. Our study calls for proper working hour management to allow for sufficient sleep, which is necessary for protecting the mental and physical health of residents.

Appropriate management of resident working schedules plays an important role in ensuring the quality of the medical services they provide. That residents can work healthily improve the quality of the medical care. It has been reported that depressed residents make 6.2 times more medication errors each month than those who are not depressed [9] and that the degree of depression and burnout is significantly negatively correlated with the quality of medical services provided by residents [12]. For the good quality of the medical services, in order to prevent depression, hospital management should adopt a systematic approach for supporting the mental health of residents while protecting them from overwork [6, 23].

The administrators of residency programs are required to improve training quality in order to meet work restrictions [29, 30]. Restricting working time could compromise the quality of training. In the United States, ACGME has mandated an 80-h working week, and a 48-h limit on work hours per week was stipulated by the Working Time Directive (2003/88/EC) in countries of the European Union [31]. In addition to positive effects such as improved resident quality of life [32] and the number of educational conferences attended [30], several studies have reported no significant differences in training quality before and after the introduction of these time restrictions, such as the monthly number of cases performed by general surgery residents [32, 33], operating room hours, and clinic time [14, 30]. Meanwhile, several negative effects were observed: a significant decrease (16.36%) in the number of patients per resident-year [30], reduced continuity of care, reduced sense of responsibility [32], reduced operative experience [34]. It’s necessary to learn clinical skills through experience on residents, so the administrators need the device which improve training quality with managing work times.

It is important not only the working hours but also the other factors for the stress of residents [35]. According to the job stress model proposed by the National Institute of Occupational Safety and Health in the United States, social support from supervisors and coworkers is a buffer factor that weakens acute reactions caused by job stressors [36]. Previous research shows that support from senior physicians and supervisors contributes to alleviating resident depression [23, 37], and early identification and intervention by a mental health care professional increases the likelihood of completing residency training [38]. Not only appropriate work management but also systematic support for mental health are important for healthy residency.

### Study limitations

This study has some limitations. First, only 251 of 853 hospitals (29.4%) participated in the study, which is a relatively small number, because participation in this study was left to the will of the individual. In addition, we only included the residents who responded to both the first and follow-up surveys. It is possible that the residents who responded to our questionnaire were interested in issues such as working conditions, mental health, or depression. However, the ratio of men and women in this study is almost the same as the ratio of official data of the country [39], there were over 1000 participants in this study and there were no significant difference in newly developed depressive symptoms between residents belonging to university hospitals and other teaching hospitals, and between male and female. Therefore, the results are thought not be influenced substantially by this circumstance. Second, we could only distribute questionnaires to residents who belonged to the limited number of hospitals that agreed to participate in this study. In addition, we only included the residents who responded to both baseline and follow-up surveys. It is possible that the residents who responded to our questionnaire were particularly interested in issues such as working conditions, mental health, or depression. In contrast, it is possible that severely depressed residents could not respond to this survey. Finally, we used a self-administered questionnaire and defined residents who had a CES-D score of 16 or more as screening-positive for depressive symptoms. Therefore, it should be noted that this algorithm may not always match a diagnosis of depression or detect residents who actually have depressive symptoms associated with depression. In a validation study of the Japanese version of the CES-D, a cut-off of 16 had a sensitivity of 88.2% and specificity of 84.8% [40]. In the present study, the prevalence of depressive symptoms was 16.0% at baseline, which was similar to the 15.2% prevalence of depression in a study in which the CES-D score was translated into Japanese. These findings suggest that the CES-D questionnaire was an effective method for screening for depressive symptoms.

## Conclusion

Resident physicians in Japan worked approximately 80 h per week. Working for more than 80 h per week was associated with a significantly higher risk of developing depression. It is important to limit the total number of working hours per week to 80 in order to maintain the health of resident physicians.

## Abbreviations

ACGME: the Accreditation Council for Graduate Medical Education
CES-D: Center for Epidemiologic Studies Depression Scale
CIs: confidence intervals
ORs: odds ratios
REIS: Residency Electronic Information System
